# Aplicações Prognósticas dos Escores Clínicos Atuais em Insuficiência Cardíaca com Fração de Ejeção Preservada: Um Estudo de Coorte Prospectivo

**DOI:** 10.36660/abc.20240852

**Published:** 2025-05-30

**Authors:** Fernando Colares Barros, Jéssica Cristina de Cezaro, Pietro Donelli Costa, Giovanni Donelli Costa, Angela Barreto Santiago Santos, Eduardo Gatti Pianca, Willian Roberto Menegazzo, Fernando Luís Scolari, Anderson Donelli da Silveira

**Affiliations:** 1 Universidade Federal do Rio Grande do Sul Porto Alegre RS Brasil Universidade Federal do Rio Grande do Sul (UFRGS), Porto Alegre, RS – Brasil; 2 Hospital de Clínicas de Porto Alegre Porto Alegre RS Brasil Hospital de Clínicas de Porto Alegre, Porto Alegre, RS – Brasil; 3 Programa de Pós-graduação em Cardiologia e Ciências Cardiovasculares UFRGS Porto Alegre RS Brasil Programa de Pós-graduação em Cardiologia e Ciências Cardiovasculares da UFRGS, Porto Alegre, RS – Brasil

**Keywords:** Diagnóstico, Insuficiência Cardíaca, Prognóstico

## Abstract

**Fundamento:**

Os escores H_2_FPEF e HFA-PEFF foram desenvolvidos para auxiliar no diagnóstico da insuficiência cardíaca com fração de ejeção preservada (ICFEP) e podem ser úteis na predição de desfechos cardiovasculares.

**Objetivo:**

Avaliar a aplicação prognóstica desses escores em uma coorte de indivíduos com ICFEP.

**Métodos:**

Este estudo prospectivo foi conduzido em um hospital terciário no Brasil entre março de 2019 e dezembro de 2021. Após a realização de exames clínicos, ecocardiograma e teste de exercício, os escores H2FPEF e HFA-PEFF foram calculados. Os pacientes foram classificados em grupos de probabilidade intermediária (H2FPEF: 2-5 pontos; HFA-PEFF: 2-4 pontos) e alta (H_2_FPEF >5 pontos; HFA-PEFF >4 pontos). O desfecho primário foi um composto de mortalidade por todas as causas e hospitalizações por ICFEP. O nível de significância estatística foi estabelecido em p<0,05.

**Resultados:**

Um total de 103 pacientes foi acompanhado por um período médio de 888 dias (±291), com idade média de 69 anos (±8,3), sendo 61% do sexo feminino. Vinte e sete pacientes (26,2%) apresentaram desfechos primários, totalizando 32 eventos, dos quais 11 foram óbitos e 21, hospitalizações por ICFEP. Na análise de curva receiver operating characteristic (ROC), o escore H_2_FPEF demonstrou melhor capacidade preditiva para os desfechos (area under the curve [AUC]: 0,637, IC 95%: 0,518-0,756, p=0,035), em comparação ao escore HFA-PEFF (AUC: 0,572, IC 95%: 0,448-0,696, p=0,270). Na análise de Kaplan-Meier, a classificação de alta probabilidade por ambos os escores foi significativamente associada à ocorrência de desfechos (log-rank p=0,034), quando comparada aos grupos com escores intermediários ou com resultados divergentes entre os dois escores.

**Conclusões:**

O escore H_2_FPEF apresentou desempenho superior ao HFA-PEFF na predição de desfechos em pacientes com ICFEP. Os resultados deste estudo contemporâneo realizado no Brasil contribuem para a estratificação de risco na prática clínica.

## Graphical abstract

**Figura Central:**
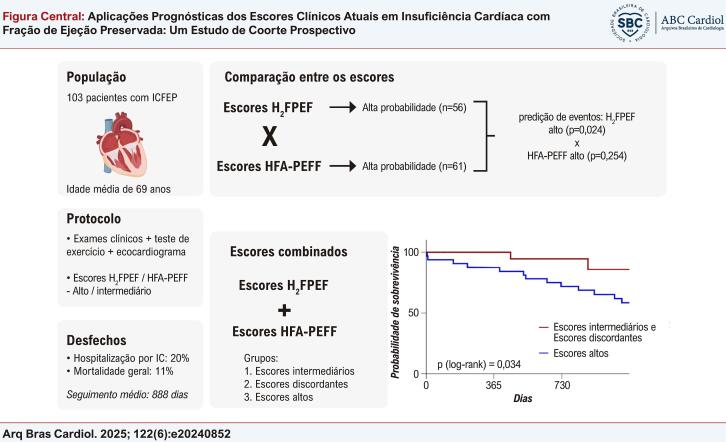
Aplicações Prognósticas dos Escores Clínicos Atuais em Insuficiência Cardíaca com Fração de Ejeção Preservada: Um Estudo de Coorte Prospectivo Aplicação prognóstica dos escores H_2_FPEF e HFA-PEFF em pacientes com ICFEP.

## Introdução

A insuficiência cardíaca (IC) com fração de ejeção preservada (ICFEP) é uma síndrome clínica prevalente, associada a altas taxas de eventos cardiovasculares e não cardiovasculares. No Brasil, a IC é a principal causa de hospitalizações e apresenta elevada taxa de reinternações, sendo que aproximadamente metade dos pacientes internados apresenta FE preservada.^1-7^

O diagnóstico da ICFEP representa um desafio clínico, em razão da alta prevalência de comorbidades nessa população e da natureza inespecífica dos sinais e sintomas, que podem ser desencadeados ou agravados por essas próprias comorbidades.^1,2^ Em pacientes com sinais e sintomas sugestivos de IC crônica, associados a fatores de risco e alterações eletrocardiográficas, o diagnóstico de ICFEP baseia-se em uma abordagem probabilística, que integra achados ecocardiográficos e a dosagem de peptídeos natriuréticos (p.ex., fragmento N-terminal do pró-peptídeo natriurético tipo B [NT-proBNP] ou peptídeo natriurético tipo B [BNP]).

Em cerca de 30% a 35% dos pacientes com ICFEP, observa-se dispneia aos esforços mesmo na ausência de sinais claros de congestão em repouso ao exame físico ou em exames de imagem.^3^ Nesses casos, a confirmação diagnóstica requer a avaliação das pressões de enchimento durante o exercício, por ecocardiograma de estresse ou cateterismo cardíaco.^3^ Contudo, essa abordagem é limitada na prática clínica por sua complexidade e alto custo, sendo geralmente restrita a centros especializados.

Nesse contexto, escores clínicos como o H_2_FPEF e o HFA-PEFF têm sido propostos como ferramentas não invasivas para auxiliar na identificação de pacientes com ICFEP.^3-9^ Baseados em modelos probabilísticos que combinam variáveis clínicas e ecocardiográficas, esses escores classificam os pacientes em baixa, intermediária ou alta probabilidade diagnóstica, orientando a necessidade de exames complementares, como ecocardiograma de estresse ou cateterismo cardíaco.

Além da aplicação diagnóstica, alguns estudos têm explorado o uso desses escores na avaliação prognóstica de pacientes com ICFEP.^5,6,9^ No caso do H_2_FPEF (acrônimo de *Heavy, Hypertensive, Atrial Fibrillation, Pulmonary Hypertension, Elder e Filling Pressures*), suas variáveis componentes estão individualmente associadas a maior risco de desfechos cardiovasculares em diferentes estudos clínicos. Da mesma forma, os componentes do HFA-PEFF (acrônimo de *Heart Failure Association Pre-test assessment, Echocardiography & natriuretic peptide, Functional testing e Final etiology*) — como massa ventricular esquerda indexada, relação E/e’, volume atrial esquerdo indexado, pressão sistólica da artéria pulmonar (PSAP) e BNP — também se associam a maior risco de eventos cardiovasculares.^6^

Estudos recentes com pacientes diagnosticados com ICFEP demonstraram que escores elevados no H_2_FPEF (>5) e no HFA-PEFF (>4) se correlacionam com maior risco de desfechos relacionados à IC.9 No entanto, há escassez de estudos prognósticos contemporâneos conduzidos no Brasil com essa população, conforme evidenciado em revisão recente.^9^

Portanto, este estudo tem como objetivo avaliar a aplicação prognóstica dos escores clínicos atuais em pacientes com ICFEP em uma coorte contemporânea no Brasil.

## Métodos

### Tipo do estudo e participantes

Este estudo de coorte prospectivo foi conduzido em um hospital universitário terciário, com pacientes diagnosticados com ICFEP. Entre março de 2019 e dezembro de 2021, os participantes foram recrutados em regime ambulatorial, sendo a maioria já em seguimento cardiológico na instituição. Foram incluídos pacientes com doença clinicamente estável há pelo menos 1 mês, em tratamento médico otimizado de acordo com as diretrizes vigentes,^1^ e com comorbidades clinicamente compensadas que não contraindicassem a realização do teste cardiopulmonar de exercício (TCPE).

### Critérios de inclusão

Os critérios diagnósticos de ICFEP foram avaliados individualmente, com base nas recomendações da literatura.^1,2^ Foram incluídos pacientes que apresentavam: i) sintomas e/ou sinais de IC; ii) fração de ejeção do ventrículo esquerdo (FEVE) preservada (>50%); iii) níveis elevados de peptídeos natriuréticos (NT-proBNP >125 pg/ml e/ou BNP >35 pg/ml) ou alterações estruturais (aumento da massa ventricular esquerda ou do volume atrial esquerdo, ambos indexados) associadas a disfunção diastólica; e iv) ausência de etiologias específicas de IC com FEVE > 50%, como miocardiopatia hipertrófica, miocardiopatia restritiva, valvopatias importantes, doenças pericárdicas, entre outras.

**Figura 1:**
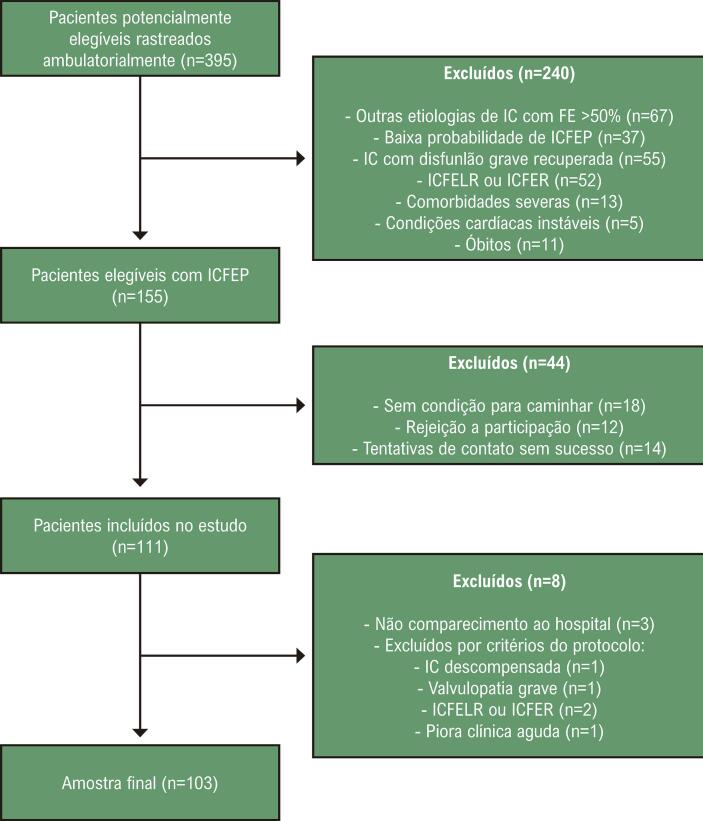
– Fluxograma de inclusão dos pacientes com ICFEP no estudo.

Para garantir uma amostra com maior probabilidade diagnóstica de ICFEP, o escore H_2_FPEF foi calculado no momento do recrutamento.4 Foram incluídos apenas os pacientes que atendiam aos critérios obrigatórios para ICFEP e que apresentavam escore H_2_FPEF alto (>5) ou intermediário (2-5), desde que com evidência de pressões de enchimento elevadas (Figura 1). Ressalta-se que o escore HFA-PEFF não foi utilizado no rastreamento, por ter sido publicado apenas em outubro de 2019.

Fonte: Elaboração própria.

### Protocolo do estudo

Os pacientes foram convidados a participar da pesquisa por meio de contato telefônico, no qual receberam uma descrição breve do estudo e tiveram agendada uma entrevista clínica com as avaliações iniciais. Nessa ocasião, foram fornecidas informações detalhadas sobre a pesquisa e assinado um termo de consentimento livre e esclarecido. Na sequência, os participantes passaram por avaliação clínica breve, incluindo medidas antropométricas e bioimpedância. A qualidade de vida foi avaliada por meio do Minnesota Living with Heart Failure Questionnaire, em versão traduzida e validada para o português por Carvalho et al.^10^ como Questionário de Qualidade de vida de Minnesota (QQVM). Em seguida, foi realizada coleta de amostras para dosagem de NT-proBNP e/ou BNP. Posteriormente, os participantes retornaram à unidade para a realização do ecocardiograma e do TCPE.

### Ecocardiograma

Imagens foram obtidas em modos bidimensionais e unidimensionais, com o uso das técnicas de Doppler colorido, contínuo, pulsátil e tecidual. Todos os exames foram realizados em aparelho Toshiba Aplio™ 300.

Foram coletadas medidas quantitativas do ventrículo esquerdo (dimensões lineares, massa ventricular esquerda e fração de ejeção pelo método de Simpson), volumes atriais e parâmetros de função diastólica (velocidades das ondas E, A, e’ septal e lateral, relação E/e’, e estimativa da PSAP). Também foram obtidas medidas lineares e de função sistólica do ventrículo direito, incluindo tricuspid annular plane systolic excursion (TAPSE), velocidade da onda S’ e fractional area change (FAC).

Todas as avaliações seguiram as recomendações da diretriz de quantificação de câmaras cardíacas da *American Society of Echocardiography*.^11^ Em pacientes com fibrilação atrial (FA), utilizaram-se as médias das velocidades de 4-5 batimentos com ciclos dentro de 20% da frequência cardíaca média e com mínima variabilidade na velocidade do fluxo mitral, para análise da função diastólica e da função sistólica do ventrículo direito.^12^

As imagens foram registradas em formato DICOM, exportadas para o prontuário eletrônico e posteriormente utilizadas para extração dos dados.

### Teste cardiopulmonar de exercício

Todos os testes foram realizados em esteira ergométrica (GE T-2100, General Electric, EUA) pelos mesmos investigadores, utilizando um protocolo de rampa com aumento progressivo de carga, individualizado para que o exame fosse concluído entre 8 e 12 minutos. Todos os participantes realizaram testes limitados por sintomas, conforme critérios de esforço máximo.

A análise dos gases foi feita com o equipamento Quark CPET (COSMED, Roma, Itália), com medições respiratórias em tempo real, acoplado ao software OMNIA (COSMED, Roma, Itália). Os limiares ventilatórios (limiar anaeróbio e ponto de compensação respiratória) foram determinados pelo método dos equivalentes ventilatórios, com confirmação do limiar anaeróbio por meio do método V-Slope.

A inclinação do equivalente VE/VCO_2_ ao longo de todo o teste e a inclinação da eficiência da captação de oxigênio (*oxygen uptake efficiency slope* [OUES]) foram calculadas. O VO_2_ pico previsto foi estimado com base no algoritmo de Wasserman e Hansen.

### Escores diagnósticos H2FPEF e HFA-PEFF

Após a conclusão dos exames do protocolo, foram calculados os escores H_2_FPEF e HFA-PEFF para todos os pacientes, bem como a probabilidade diagnóstica de ICFEP conforme cada critério.

**Figura 2:**
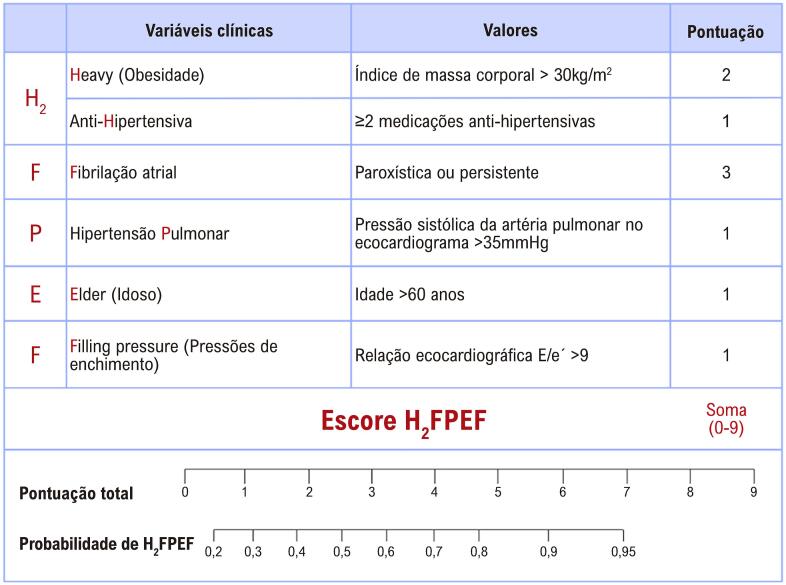
– Escore H2FPEF no diagnóstico de ICFEP e estimativa de probabilidade diagnóstica.

O escore H_2_FPEF combina variáveis clínicas e ecocardiográficas, incluindo: obesidade (*Heavy*), uso de dois ou mais anti-hipertensivos (*Hypertensive*), FA (*Atrial Fibrillation*), PSAP >35 mmHg (*Pulmonary Hypertension*), idade acima de 60 anos (*Elder*) e relação E/e’ >9 (*Filling Pressures*). Os pacientes foram classificados conforme a pontuação diagnóstica: >5 pontos (alta probabilidade), 2 a 5 pontos (probabilidade intermediária) e 1 ponto (baixa probabilidade) (Figura 2).

Fonte: Adaptado de Reddy et al.4

**Figura 3:**
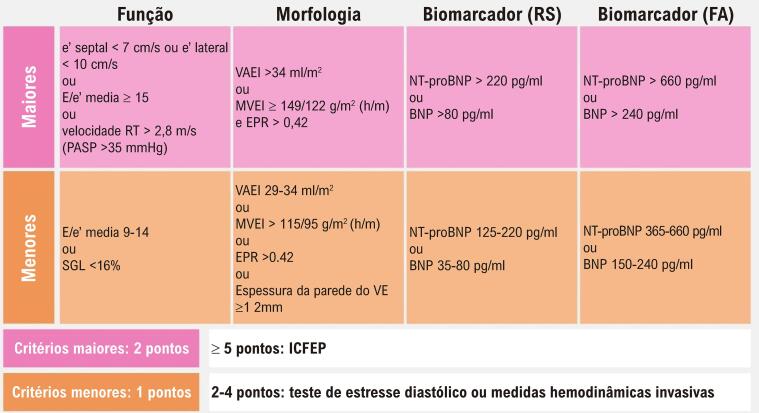
– Escore HFA-PEFF no diagnóstico de ICFEP e estimativa da probabilidade de doença. Fonte: Adaptado de Pieske B, et al. How to diagnose heart failure with preserved ejection fraction: the HFA-PEFF diagnostic algorithm: a consensus recommendation from the Heart Failure Association (HFA) of the European Society of Cardiology (ESC).^7^

Para o cálculo do escore HFA-PEFF, foram utilizadas variáveis ecocardiográficas dos domínios morfológico e funcional, além dos níveis de peptídeos natriuréticos (NT-proBNP ou BNP), estratificadas em critérios maiores (2 pontos) e menores (1 ponto). Os pacientes foram classificados conforme a pontuação total: >4 pontos (alta probabilidade), 2-4 pontos (probabilidade intermediária) e 1 ponto (baixa probabilidade) (Figura 3).

### Desfecho primário e seguimento

O desfecho primário foi definido como um composto de mortalidade por todas as causas e hospitalizações por IC. Os pacientes foram acompanhados prospectivamente por um período mínimo de 2 anos e máximo de 3 anos, por meio de i) busca ativa nos registros médicos, com revisão de consultas ambulatoriais e internações, e ii) contatos telefônicos periódicos com os participantes para identificação de desfechos de interesse. As avaliações individuais dos desfechos ocorreram a cada 6 meses, e os resultados foram registrados em um formulário padronizado e transferidos para o software REDCap.^13^

### Análise estatística

Foi realizado cálculo de tamanho amostral para detectar um efeito significativo do consumo de oxigênio de pico (VO_2_ pico), categorizado como acima ou abaixo da mediana, em modelo de regressão de Cox. Consideraram-se grupos de tamanhos iguais, com as seguintes estimativas: taxa de desfechos primários de 8,83% em pacientes com VO_2_ >17,1 ml/kg/min e de 31,17% naqueles com VO_2_ <17,1 ml/kg/min, com hazard ratio (HR) de 3,53, em um seguimento de 2 anos.^14^ Utilizou-se a função “ssizeCT.default” do pacote “powerSurvEpi” no software R versão 3.5.0, considerando poder estatístico de 80% e nível de significância de 5%. O cálculo estimou a necessidade de 126 pacientes.

As análises estatísticas foram realizadas no software SPSS, versão 29.0 (IBM SPSS Statistics, Chicago, IL, EUA). A normalidade das variáveis foi avaliada pelo teste de Shapiro-Wilk. Variáveis contínuas com distribuição normal foram comparadas por meio do teste t para amostras independentes, enquanto variáveis não normalmente distribuídas foram analisadas pelo teste de Mann-Whitney. Para variáveis categóricas, utilizou-se o teste do qui-quadrado.

Os resultados foram expressos como média±desvio padrão ou mediana com intervalos interquartis para variáveis contínuas, e como frequências absolutas e relativas para variáveis categóricas. A regressão de riscos proporcionais de Cox foi utilizada para avaliar o impacto independente e ajustado das variáveis no prognóstico, em modelo multivariado. A análise de curva receiver operating characteristic (ROC) foi utilizada para comparar a capacidade discriminatória dos escores na predição de desfechos. A análise de sobrevida foi realizada com regressão de Cox multivariada e representada por curvas de Kaplan-Meier. A concordância diagnóstica entre os escores foi avaliada por meio da estatística Kappa. Valores de p<0,05 foram considerados estatisticamente significativos.

## Resultados

A amostra final incluiu 103 pacientes, com idade média de 69,1 anos (±8,3), sendo a maioria do sexo feminino (61,2%). Entre os participantes, 57 (55,3%) apresentavam hospitalização prévia por IC, e 82 (79,6%) estavam em classe funcional de acordo com a *New York Heart Association* (NYHA) I ou II, enquanto 21 (20,4%) estavam em classe III. O tempo médio de seguimento foi de 888 dias (±291). A Figura Central apresenta uma síntese dos principais achados do estudo.

Após a realização dos exames do protocolo, foram calculados os escores H_2_FPEF e HFA-PEFF, e os pacientes foram classificados em grupos de probabilidade diagnóstica intermediária ou alta para ICFEP. Pelo escore H_2_FPEF, 56 pacientes (54,3%) apresentaram alta probabilidade e 47 (45,7%) probabilidade intermediária. Pelo escore HFA-PEFF, 61 (59,2%) apresentaram alta probabilidade, 41 (39,8%) probabilidade intermediária e 1 (1,0%) baixa probabilidade. Considerando a combinação dos escores, 32 pacientes (31,1%) apresentaram alta probabilidade por ambos os critérios, 53 (51,5%) apresentaram resultados discordantes, e 18 (17,5%) foram classificados como probabilidade intermediária por ambos. A análise de concordância entre os escores revelou valor de Kappa de -0,036 (p=0,718).

As características gerais da amostra, estratificadas segundo os escores H2FPEF e HFA-PEFF (probabilidade intermediária ou alta), estão apresentadas nas Tabelas 1 e 2. Os valores são expressos como médias, medianas ou frequências relativas, conforme apropriado.

**Tabela 1:** – Características clínicas e antropométricas conforme os escores H2FPEF e HFA-PEFF

Variável	H^2^FPEF intermediário (n=47)	H^2^FPEF alto (n=56)	Valor p	HFA-PEFF intermediário (n=41)	HFA-PEFF alto (n=61)	Valor p
**Idade (anos)**	68,6 (8,4)	69,4 (8,3)	0,632	69,0 (7,4)	69,4 (8,6)	0,804
**Sexo feminino, n (%)**	33 (70%)	30 (54%)	0,106	27 (66%)	36 (59%)	0,537
**Hospitalizações por IC, n (%)**	14 (30%)	43 (77%)	<0,001	26 (63%)	30 (49%)	0,223
**Comorbidades, n (%)**						
Obesidade	36 (77%)	42 (75%)	1,000	33 (80%)	44 (72%)	0,360
Hipertensão	47 (100%)	53 (95%)	0,248	39 (95%)	60 (98%)	0,563
Diabetes melito	30 (64%)	28 (50%)	0,170	23 (56%)	35 (57%)	1,000
FA ou flutter atrial	3 (6%)	46 (82%)	<0,001	19 (46%)	29 (47%)	1,000
Doença coronariana	19 (40%)	18 (32%)	0,415	12 (29%)	25 (41%)	0,295
Doença renal crônica	20 (43%)	22 (39%)	0,841	18 (44%)	24 (39%)	0,685
**Hábitos de vida, n (%)**						
Atividade física	15 (32%)	12 (21%)	0,265	7 (17%)	19 (31%)	0,164
Tabagismo	26 (55%)	34 (61%)	0,689	23 (56%)	36 (59%)	0,839
**Escore QQVM**	33,2 (22,8)	33,4 (20,2)	0,969	35,9 (21,2)	31,5 (21,5)	0,325
**Medicações em uso, n (%)**						
Betabloqueadores	43 (91%)	46 (82%)	0,249	35 (85%)	53 (87%)	1,000
Inibidores de ECA/BRA	36 (77%)	47 (84%)	0,454	33 (80%)	49 (80%)	1,000
Espironolactona	8 (17%)	8 (14%)	0,788	6 (15%)	9 (15%)	1,000
Diurético de alça	22 (47%)	39 (70%)	0,027	23 (56%)	37 (61%)	0,685
**Exames laboratoriais**						
Clearance de creatinina (ml/min)	62,7 (19,4)	58,1 (17,8)	0,220	59,0 (20,9)	60,6 (16,9)	0,668
Hemoglobina (g/dl)	12,9 (1,4)	13,1 (1,6)	0,550	12,8 (1,5)	13,2 (1,5)	0,296
**Peptídeos natriuréticos**						
NT-proBNP, mediana (pg/ml) (n=55)	225,7 (152,0-326,6)	695,1 (329,4-1.725,0)	<0,001	158,6 (121,2-308,3)	447,6 (246,4-1.318,2)	0,011
BNP, mediana (pg/ml) (n=28)	86,9 (31,5-123,9)	147,4 (118,1-273,2)	0,002	70,2 (23,5-144,0)	126,8 (89,2-190,4)	0,035
**Medidas antropométricas**						
IMC (kg/m^2^)	34,0 (5,7)	33,3 (5,3)	0,529	34,4 (6,0)	33,1 (5,1)	0,249
Massa magra (%)	58,2 (8,5)	62,2 (12,1)	0,054	58,9 (10,6)	61,3 (10,9)	0,277

Para variáveis contínuas, utilizaram-se os testes t de Student ou de Mann-Whitney, conforme a distribuição dos dados. Para variáveis categóricas, utilizou-se o teste do qui-quadrado. As medidas de dispersão são apresentadas entre parênteses como DP ou IIQ, conforme apropriado. BRA: bloqueador dos receptores de angiotensina; DP: desvio padrão; FA: fibrilação atrial; IECA: inibidor da enzima conversora de angiotensina; IIQ: intervalo interquartil; IMC: índice de massa corporal; QQVM: Questionário de Qualidade de Vida de Minnesota.

Fonte: Elaboração própria.

**Tabela 2:** – Resultados do ecocardiograma e do teste cardiopulmonar de exercício segundo os escores H2FPEF e HFA-PEFF

Variável	H^2^FPEF intermediário (n=47)	H^2^FPEF alto (n=56)	Valor p	HFA-PEFF intermediário (n=41)	HFA-PEFF alto (n=61)	Valor p
**Ecocardiograma**						
MVE (g/m^2^)	92,4 (24,0)	98,2 (22.1)	0,207	90,8 (21,6)	98,9 (23,7)	0,082
Espessura da parede septal (mm)	10,5 (1,5)	10,9 (1,5)	0,174	10,7 (1,5)	10,7 (1,6)	0,804
Espessura da parede posterior (mm)	10,0 (1,4)	10,3 (1,6)	0,311	10,0 (1,5)	10,3 (1,5)	0,365
EPR	0,43 (0,07)	0,43 (0,07)	0,761	0,44 (0,08)	0,43 (0,06)	0,624
Fração de ejeção (%)	61,5 (4,4)	58,4 (4,3)	0,001	58,9 (5,2)	60,5 (4,0)	0,090
Volume do AE indexado (ml/m2)	41,8 (9,8)	53,8 (16,0)	<0,001	45,3 (12,8)	50,8 (15,4)	0,061
Volume do AD indexado (ml/m2)	31,1 (15,2)	44,4 (20,9)	<0,001	34,5 (14,7)	41,5 (22,4)	0,082
Diâmetro basal do VD (mm)	36,5 (3,8)	39,4 (5,3)	0,002	38,8 (4,6)	37,4 (5,0)	0.157
TAPSE (mm)	20,2 (3,9)	17,3 (4,0)	<0,001	17,9 (4,2)	19,1 (4,1)	0,147
FAC (%)	44,4 (5,6)	42,2 (5,8)	0,069	43,1 (6,6)	43,3 (5,2)	0.879
PSAP (mmHg)	28,6 (5,1)	37,3 (10,7)	<0,001	32,9 (8,6)	35,2 (10,8)	0,310
Pressão venosa central (mmHg)	4,5 (2,3)	6,9 (4,3)	<0,001	5,8 (3,4)	5,8 (4,0)	0,974
Onda e’ septal (cm/s)	5,7 (1,4)	6,9 (2,0)	0,001	6,6 (2,3)	6,2 (1,5)	0,310
Onda e’ lateral (cm/s)	7,1 (1,8)	9,3 (2,9)	<0,001	9,0 (2,7)	7,8 (2,5)	0,020
Relação E/e’	13,1 (4,7)	13,2 (6,2)	0,926	12,5 (5,6)	13,6 (5,4)	0,323
**Teste cardiopulmonar de exercício**						
FC pico (% da máxima prevista)	81,5 (12,3)	85,7 (17,1)	0,159	87,0 (13,6)	81,3 (15,7)	0,071
VO_2_ pico relativo (ml/kg/min)	15,1 (2,5)	14,7 (3,2)	0,465	14,4 (2,9)	15,1 (2,9)	0,245
VO_2_ pico (% do previsto)	80,5 (14,8)	74,0 (15,2)	0,033	75,4 (12,9)	78,2 (16,8)	0,380
Inclinação VE/VCO_2_	33,2 (7,7)	38,5 (8,8)	0,002	35,7 (9,0)	36,3 (8,6)	0,723
VE/VCO_2_ pico	32,5 (6,5)	36,9 (5,5)	0,001	34,9 (7,0)	34,6 (6,0)	0,827
PetCO_2_ (mmHg)	32,4 (4,4)	30,8 (4,2)	0,085	32,5 (4,2)	30,9 (4,4)	0,088

O teste t de Student foi utilizado para variáveis contínuas. Os valores são apresentados como média (DP). AD: átrio direito; AE: átrio esquerdo; DP: desvio padrão; EPR: espessura parietal relativa; FAC: fractional area change; FC: frequência cardíaca; MVE: massa ventricular esquerda; PSAP: pressão sistólica da artéria pulmonar; TAPSE: tricuspid annular plane systolic excursion; VD: ventrículo direito; VO2: consumo de oxigênio.

Fonte: Elaboração própria.

### Escores diagnósticos H2FPEF e HFA-PEFF

Os resultados estão apresentados na Tabela 3. Em 20 pacientes (19,4%) não foi possível estimar tecnicamente a PSAP. Em outros 20 pacientes (19,4%), não houve coleta de peptídeos natriuréticos devido a alterações no protocolo institucional, que só foi normalizado próximo ao início da pandemia, inviabilizando a coleta em tempo hábil.

**Tabela 3:** – Variáveis clínicas e ecocardiográficas atribuídas pelos escores H2FPEF e HFA-PEFF

Variáveis	Escore intermediário	Escore alto	Valor p
**Escore H_2_FPEF (n=103)**	47 (46%)	56 (54%)	
Total de pontos	4,3 (0,9)	7,3 (1,1)	<0,001
Obesidade	35 (74%)	42 (75%)	1
Uso de >2 anti-hipertensivos	44 (94%)	49 (87%)	0,339
FA	3 (6%)	46 (82%)	<0,001
Idade >60 anos	38 (81%)	49 (87%)	0,419
Relação E/e’ >9	40 (85%)	40 (71%)	0,153
PSAP >35 mmHg	3 (6%)	32 (57%)	<0,001
PSAP indisponível	16 (34%)	4 (7%)	0,001
**Escore HFA-PEFF (n=102)**	41 (40%)	61 (59%)	
Total de pontos	3,6 (0,7)	5,7 (0,5)	<0,001
Domínio funcional*			
E’ septal <7 cm/s	27 (66%)	44 (72%)	0,518
E’ lateral <10 cm/s	29 (71%)	49 (80%)	0,342
E/e’ média >15	9 (22%)	19 (31%)	0,369
E/e’ média 9-14	20 (49%)	32 (52%)	0,840
Velocidade da RT >2,8 m/s	13 (32%)	23 (38%)	0,673
Velocidade da RT indisponível	5 (12%)	15 (25%)	0.137
**Domínio morfológico**			
Volume do AE >34ml/m^2^	34 (83%)	58 (95%)	0,085
Volume do AE 29-34ml/m^2^	3 (7%)	3 (5%)	0,682
MVE >149/122 g/m^2^ (H/M) e EPR >0,42	1 (2%)	5 (8%)	0,397
MVE >115/95 g/m^2^ (H/M)	10 (24%)	22 (36%)	0,278
EPR >0,42	19 (46%)	34 (56%)	0,420
Espessuras parietais >12 mm	8 (19%)	17 (28%)	0,360
**Peptídeos natriuréticos**			
NT-proBNP >220 / BNP >80 (sem FA)	0 (0%)	33 (54%)	<0,001
NT-proBNP 125-220 / BNP 35-80 (sem FA)	2 (5.0%)	9 (15%)	0,192
NT-proBNP >660 / BNP >240 (com FA)	2 (5,0%)	13 (21%)	0,024
NT-proBNP 365-660 / BNP 105-240 (com FA)	1 (2%)	6 (10%)	0,237
Não preencheu critérios	16 (39%)	0 (0%)	<0,001
Peptídeo não coletado	20 (49%)	0 (0%)	<0,001

*A variável strain global longitudinal (SGL) não foi incluída neste estudo. O teste qui-quadrado foi utilizado para as variáveis categóricas. AE: átrio esquerdo; BNP: peptídeo natriurético tipo B; EPR: espessura parietal relativa; FA: fibrilação atrial; H/M: homens/mulheres; MVE: massa ventricular esquerda; PSAP: pressão sistólica da artéria pulmonar; RT: regurgitação tricúspide.

Fonte: Elaboração própria.

### Desfechos primários

Dos 103 pacientes, 27 (26,2%) apresentaram desfechos primários, totalizando 32 eventos, sendo 11 óbitos e 21 hospitalizações por IC. Entre os óbitos, quatro ocorreram por complicações neoplásicas, três por sepse respiratória, três por causas cardiovasculares e um por COVID-19. O tempo médio até o primeiro evento foi de 563 dias (± 356). Os resultados estão apresentados na Tabela 4.

**Tabela 4:** – Desfechos primários e escores prognósticos segundo os escores H2FPEF e HFA-PEFF

Variável	ICFEP total (n=103)	H_2_FPEF intermediário (n=47)	H_2_FPEF alto (n=56)	Valor p	HFA-PEFF intermediário (n=41)	HFA-PEFF alto (n=61)	Valor p
**Pacientes com desfechos primários, n (%)**	27 (26%)	7 (15%)	20 (36%)	0,024	8 (19%)	19 (31%)	0,254
Escore H2FPEF	6,5 (1,6)	4,4 (0,8)	7,3 (1,1)		6,6 (1,7)	6,5 (1,6)	
Escore HFA-PEFF	5,2 (1,0)	5,4 (1,0)	5,1 (1,1)		3,8 (0,5)	5,8 (0,4)	
**Desfechos primários, n**	32	10	22		9	23	
Morte, n (%)	11 (11%)	5 (11%)	6 (11%)	0,990	3 (7,3%)	8 (13%)	0,518
Hospitalizações por IC, n (%)	21 (20%)	5 (11%)	16 (29%)	0,029	6 (15%)	15 (25%)	0,318
Escore MAGGIC (média±DP)	17,3 (4,7)	16,9 (4,5)	17,7 (5,0)	0,420	17,3 (4,6)	17,5 (4,7)	0,800

Os percentuais entre parênteses referem-se ao total de pacientes (n) em cada coluna. DP: desvio padrão; IC: insuficiência cardíaca; ICFEP: insuficiência cardíaca com fração de ejeção preservada; MAGGIC: Meta-Analysis Global Group in Chronic Heart Failure.

Fonte: Elaboração própria.

Pacientes com escore alto no H_2_FPEF apresentaram maior frequência de desfechos primários em comparação àqueles com escore intermediário (35,7% vs 14,9%; p<0,024), assim como maior ocorrência de hospitalizações por IC (29,0% vs 11,0%; p<0,029). Em relação ao escore HFA-PEFF, não houve diferença estatisticamente significativa entre os grupos de probabilidade intermediária e alta.

Quando combinados os critérios, pacientes com escores altos tanto no H_2_FPEF quanto no HFA-PEFF apresentaram maior frequência de desfechos em comparação àqueles com escores discordantes ou com ambos intermediários (43,8% vs 20,7% vs 11,1%; p<0,007). Os resultados estão apresentados na Tabela 5.

**Tabela 5:** – Desfechos primários conforme os escores combinados H2FPEF e HFA-PEFF

Variáveis	ICFEP total (n=103)	Ambos intermediários (n=18)	Escores discordantes (n=53)	Ambos altos (n=32)	Valor p
**Pacientes com desfecho primário, n (%)**	27 (26%)	2 (11%)	11 (20,7%)	14 (44%)	<0.007
Escore H2FPEF	6,5 (1,6)	4,5 (0,7)	6,0 (1,84)	7.3 (1,1)	
Escore HFA-PEFF	5,2 (1,0)	4,0 (0,0)	4,7 (1,3)	5.7 (0,4)	
**Desfechos primários, n**	32	3	13	16	
Morte, n (%)	11 (11%)	2 (11%)	4 (7,5%)	5 (16%)	0.491
Hospitalizações por IC, n (%)	21 (20%)	1 (6%)	9 (17,0%)	11 (34%)	0.012

Os percentuais entre parênteses referem-se ao total de pacientes (n) em cada coluna. IC: insuficiência cardíaca; ICFEP: insuficiência cardíaca com fração de ejeção preservada.

Fonte: Elaboração própria.

### Preditores de desfechos

Na análise de regressão de Cox, no modelo univariado, o escore H_2_FPEF alto não foi estatisticamente significativo na predição de eventos (HR: 2,316; IC 95%: 0,973-5,513; p=0,058), assim como o escore HFA-PEFF alto (HR: 1,570; IC 95%: 0,683-3,611; p=0,288). A presença concomitante de escores altos no H2FPEF e no HFA-PEFF também não se associou de forma significativa à predição de desfechos (HR: 3,850; IC 95%: 0,868-17,071; p=0,076).

**Figura 4:**
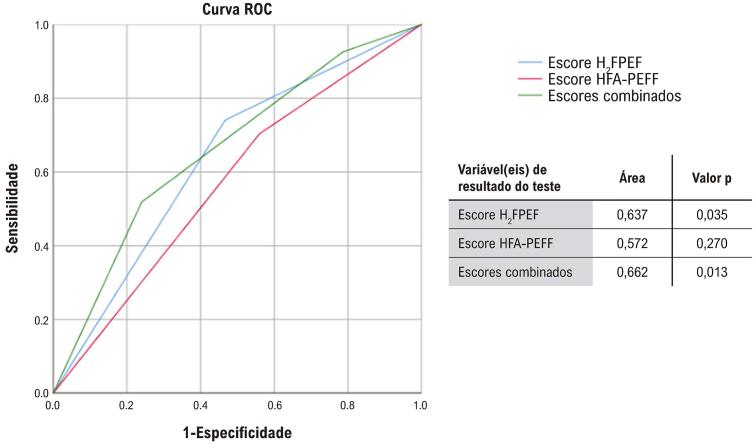
– Curvas ROC dos escores H2FPEF, HFA-PEFF e combinados na predição de desfechos.

Na análise das curvas ROC, o escore H_2_FPEF apresentou melhor capacidade preditiva de desfechos (AUC: 0,637; IC 95%: 0,518-0,756; p=0,035), em comparação ao escore HFA-PEFF (AUC: 0,572; IC 95%: 0,448-0,696; p=0,270). Quando os escores foram combinados, observou-se um incremento na capacidade discriminatória para predição de desfechos (AUC: 0,662; IC 95%: 0,543-0,782; p=0,013). Os resultados estão apresentados na Figura 4.

Fonte: Elaboração própria.

**Figura 5:**
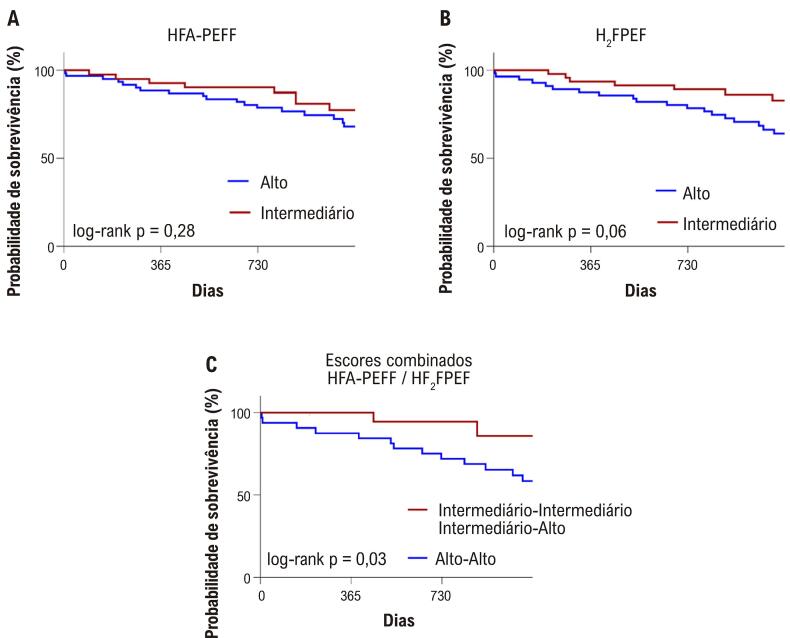
– Curvas de sobrevida da análise de Kaplan-Meier segundo os escores HFA-PEFF (A), H2FPEF (B) e escores combinados (C).

Na análise de sobrevida pelo método de Kaplan-Meier, o escore H_2_FPEF alto apresentou uma tendência à significância estatística na predição de desfechos (log-rank p=0,05), enquanto o escore HFA-PEFF não foi significativo (log-rank p=0,284). A presença concomitante de escores altos em ambos os critérios foi significativamente associada à ocorrência de desfechos (log-rank p=0,034), em comparação ao conjunto dos demais pacientes (escores discordantes e ambos intermediários). A Figura 5 apresenta as curvas de sobrevida obtidas pela análise de Kaplan-Meier, conforme cada critério avaliado.

Fonte: Elaboração própria.

## Discussão

**Figura 6:**
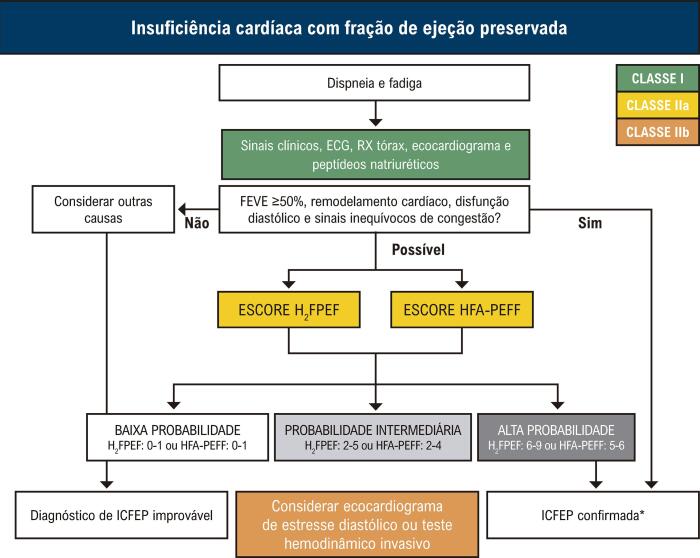
Modelo atual de diagnóstico probabilístico da ICFEP.

Este estudo teve como objetivo avaliar o impacto prognóstico dos escores clínicos H_2_FPEF e HFA-PEFF em uma amostra bem caracterizada de pacientes com ICFEP no Brasil. Ambos os escores foram desenvolvidos para auxiliar no diagnóstico não invasivo da ICFEP, estratificando os pacientes em baixa, intermediária e alta probabilidade diagnóstica (Figura 6). Os resultados demonstraram que o escore H_2_FPEF apresentou desempenho superior ao HFA-PEFF na predição de desfechos cardiovasculares em uma coorte prospectiva de pacientes com ICFEP. Além disso, a combinação de ambos os escores esteve associada a um risco aumentado de eventos cardiovasculares. Considerando a elevada prevalência da ICFEP no Brasil, esses achados têm importância clínica, pois permitem a identificação não invasiva de pacientes com maior risco de eventos cardiovasculares graves.

Fonte: Marcondes-Braga FG et al. Emerging Topics Update of the Brazilian Heart Failure Guideline – 2021.^8^

O escore H_2_FPEF foi desenvolvido com base em uma coorte de pacientes com diagnóstico invasivo de ICFEP, confirmado por cateterismo cardíaco combinado com exercício físico, e posteriormente validado em uma segunda coorte composta por pacientes com e sem ICFEP.4 As variáveis clínicas incluídas no escore — obesidade, FA, uso de dois ou mais anti-hipertensivos, idade acima de 60 anos, relação E/e’ >9 e PSAP >35 mmHg — demonstraram ser preditoras de desfechos cardiovasculares em modelo multivariado. Escores H_2_FPEF elevados (>5) foram associados a uma probabilidade diagnóstica de ICFEP superior a 90%. Por sua vez, o escore HFA-PEFF foi desenvolvido a partir de um consenso da European Society of Cardiology e baseia-se em uma abordagem diagnóstica sequencial em quatro etapas: i) avaliação inicial pré-teste; ii) avaliação probabilística baseada em achados ecocardiográficos e nos níveis de peptídeos natriuréticos; iii) exames complementares especializados; e iv) avaliação etiológica.7 Na segunda etapa do fluxograma, os valores de corte das variáveis ecocardiográficas (domínios morfológico e funcional) e dos níveis de NT-proBNP/BNP foram definidos com base em suas sensibilidades e especificidades para o diagnóstico de ICFEP, conforme evidências de estudos clínicos. Essas variáveis foram classificadas como critérios maiores (2 pontos) ou menores (1 ponto). Escores HFA-PEFF superiores a 4 pontos foram definidos pelo consenso como indicativos de alta probabilidade diagnóstica de ICFEP. Diferentemente do escore H_2_FPEF, os critérios do HFA-PEFF não foram inicialmente validados em uma coorte composta por pacientes com e sem ICFEP.7,15,16

Refletindo a realidade clínica dos pacientes com ICFEP, nossa amostra foi predominantemente composta por indivíduos mais idosos, do sexo feminino, com obesidade, hipertensão, diabetes, doença renal crônica e doença coronariana. A prevalência de FA e de outras alterações cardíacas — como hipertrofia ventricular esquerda, disfunção diastólica, aumento do átrio esquerdo, hipertensão pulmonar e disfunção do ventrículo direito — esteve em conformidade com uma revisão clínica recente sobre pacientes com ICFEP.^3^

No presente estudo, no início da fase de inclusão (março de 2019), o escore H_2_FPEF foi utilizado para estimar de forma não invasiva a probabilidade diagnóstica de ICFEP, sendo incluídos apenas pacientes com escore alto ou com escore intermediário associado a sinais de aumento das pressões de enchimento. A amostra foi composta por uma proporção significativa de pacientes com alta probabilidade diagnóstica: 54% pelo escore H2FPEF e 59% pelo HFA-PEFF. Esses percentuais são superiores aos observados em estudos prognósticos recentes que também analisaram a aplicação desses escores. No estudo de Przewlocka-Kosmala et al.,^5^ 30% dos pacientes apresentavam escore H_2_FPEF >5 e 41% dos pacientes apresentavam escore HFA-PEFF >4. Já no estudo de Egashira et al.,6 38% apresentavam escore HFA-PEFF >4.

Entre os grupos de escore intermediário e alto segundo o critério HFA-PEFF, a única diferença estatisticamente significativa foi observada nos níveis de peptídeos natriuréticos. O mesmo padrão foi identificado na estratificação pelo escore H_2_FPEF, apesar de esse parâmetro não compor seu cálculo. De acordo com estudo recente de Reddy et al.,^17^ as diferenças de acurácia diagnóstica entre os escores H_2_FPEF e HFA-PEFF podem estar relacionadas aos componentes primários de cada modelo. No H_2_FPEF, predominam variáveis clínicas como obesidade, hipertensão e FA, que elevam a probabilidade pré-teste de ICFEP. Já o HFA-PEFF baseia-se em alterações ecocardiográficas e nos níveis de peptídeos natriuréticos, parâmetros com menor sensibilidade para o diagnóstico, embora informativos sobre a presença da doença. Em pacientes com FA, o escore HFA-PEFF utiliza valores mais elevados de peptídeos natriuréticos para confirmar o diagnóstico de ICFEP.^17^

No presente estudo, observou-se maior taxa de desfechos primários entre os pacientes com alta probabilidade diagnóstica em comparação àqueles com probabilidade intermediária, com significância estatística apenas para o escore H_2_FPEF e para a combinação dos escores. A estratificação dos pacientes em probabilidade intermediária e alta pelo escore HFA-PEFF esteve limitada à etapa 2 do fluxograma proposto pelo consenso, uma vez que não foram realizadas medidas ecocardiográficas durante o exercício (etapa 3). Conforme demonstrado por Przewlocka-Kosmala et al.,^5^ o uso da ecocardiografia sob estresse pode aumentar a capacidade preditiva do escore HFA-PEFF.

Na presente amostra, a combinação de escores de alta probabilidade proporcionou um incremento na identificação de desfechos cardiovasculares. No entanto, conforme demonstrado em outros estudos,^5-7,18-20^ houve discordância significativa na estimativa de probabilidade diagnóstica quando os escores foram utilizados em conjunto, com divergência observada em 51% dos pacientes, concordância em 31% entre aqueles com escores altos e em apenas 17% entre os com escores intermediários. Essa variabilidade compromete a aplicabilidade clínica da abordagem combinada na prática assistencial.

Nosso estudo apresenta algumas limitações. A principal delas foi o início da pandemia de COVID-19 durante o período de inclusão, que precisou ser interrompido em março de 2020 e foi retomado apenas em abril de 2021, de forma mais restrita, em razão das medidas de isolamento e prevenção. Essa interrupção inviabilizou o alcance da amostra prevista (n=126), mesmo com a extensão do período de inclusão por mais 18 meses. Apesar disso, todos os pacientes incluídos (n=103) completaram o seguimento mínimo de 2 anos e máximo de 3 anos, conforme estabelecido no protocolo. Houve apenas um óbito relacionado à infecção por COVID-19, de modo que a generalização dos resultados não foi comprometida. É possível que alguns desfechos adicionais não tenham sido registrados, devido ao acompanhamento incompleto de uma pequena parte da amostra (n=5). Em segundo lugar, durante o estudo, houve uma alteração institucional no tipo de peptídeo natriurético utilizado (de NT-proBNP para BNP), o que, até a normalização da coleta — pouco antes do início da pandemia —, resultou na ausência desse dado em 20 pacientes. Como os níveis de BNP/NT-proBNP são utilizados no cálculo do escore HFA-PEFF, essa limitação pode ter impactado a acurácia na estratificação entre os grupos de risco intermediário e alto. Em terceiro lugar, em 20 pacientes (19,4%) não foi possível estimar a PSAP via ecocardiografia, critério incluído no escore H_2_FPEF. Embora esse percentual seja inferior ao observado em outros estudos (30-40%),^18,21,22^ a indisponibilidade desse dado pode ter gerado imprecisão na classificação entre risco intermediário e alto nesse escore. Ainda assim, nossa amostra apresentou percentuais semelhantes de pacientes com alta probabilidade diagnóstica segundo ambos os escores (54% para H_2_FPEF e 59% para HFA-PEFF), o que permitiu comparações consistentes entre os grupos. Em quarto lugar, a ausência da modalidade de ecocardiografia sob estresse pode ter limitado a capacidade preditiva do escore HFA-PEFF, uma vez que não foram observadas diferenças significativas entre os grupos de risco intermediário e alto pela etapa 2 do fluxograma diagnóstico. Entretanto, essa modalidade não estava disponível no nosso hospital, possui custo elevado e é restrita a poucos centros especializados, o que também limita sua aplicabilidade na prática clínica. Por fim, trata-se de um estudo conduzido em centro único, o que pode restringir a generalização dos achados para outras populações. No entanto, a coorte analisada é representativa de pacientes com ICFEP, conforme descrito em uma revisão clínica recente.^3^

Entre as principais forças do presente estudo, destaca-se, em primeiro lugar, o delineamento prospectivo de coorte, com inclusão de pacientes com base em critérios clínicos específicos e atualizados para o diagnóstico de ICFEP, refletindo de forma fiel a população acometida pela doença. Em segundo lugar, ressalta-se a aplicação de um protocolo clínico não invasivo direcionado à ICFEP, que incluiu avaliação clínica breve, medidas antropométricas e de bioimpedância, coleta de peptídeo natriurético, ecocardiograma e teste de exercício. Esse protocolo favorece a comparação com outras populações e amplia o potencial de generalização dos achados. Em terceiro lugar, diante da escassez de estudos prognósticos contemporâneos com pacientes com ICFEP no Brasil, os resultados obtidos assumem relevância para a prática clínica nacional.

## Conclusões

Em uma coorte prospectiva de pacientes com ICFEP no Brasil, o escore H_2_FPEF apresentou desempenho superior ao escore HFA-PEFF na predição de desfechos cardiovasculares. A combinação de ambos os escores, quando em alta probabilidade, proporcionou maior capacidade prognóstica para eventos cardiovasculares. Considerando a elevada prevalência da ICFEP no país, esses achados têm relevância clínica ao possibilitar, de forma não invasiva, a identificação de pacientes com maior risco de desfechos cardiovasculares graves.
