# Supplementary material for: Aplicações Prognósticas dos Escores Clínicos Atuais em Insuficiência Cardíaca com Fração de Ejeção Preservada: Um Estudo de Coorte Prospectivo
Source: Arq Bras Cardiol. 2025 May 30;122(6):e20240852. [Article in Portuguese] doi: 10.36660/abc.20240852 (PMC12212386; doi:10.36660/abc.20240852)
Supplement: Translation

# Translation: Prognostic Applications of Current Clinical Scores in Heart Failure with Preserved Ejection Fraction: A Prospective Cohort Study

**DOI:** 10.36660/abc.20240852i

**Authors:** Fernando Colares Barros, Jéssica Cristina de Cezaro, Pietro Donelli Costa, Giovanni Donelli Costa, Angela Barreto Santiago Santos, Eduardo Gatti Pianca, Willian Roberto Menegazzo, Fernando Luís Scolari, Anderson Donelli da Silveira

**Affiliations:** 1 Brazil Universidade Federal do Rio Grande do Sul (UFRGS), Porto Alegre, RS – Brazil; 2 Brazil Hospital de Clínicas de Porto Alegre, Porto Alegre, RS – Brazil; 3 Brazil Programa de Pós-graduação em Cardiologia e Ciências Cardiovasculares da UFRGS, Porto Alegre, RS – Brazil

**Keywords:** Diagnosis, Heart Failure, Prognosis

## Abstract

**Background:**

The H2FPEF and HFA-PEFF scores were developed to support the diagnosis of heart failure with preserved ejection fraction (HFpEF) and may also help predict cardiovascular outcomes.

**Objective:**

To assess the prognostic value of these scores in a cohort of individuals with HFpEF.

**Methods:**

This prospective study was conducted at a tertiary hospital in Brazil between March 2019 and December 2021. After clinical evaluation, echocardiography, and exercise testing, the H2FPEF and HFA-PEFF scores were calculated. Patients were classified into intermediate probability groups (H_2_FPEF: 2-5 points; HFA-PEFF: 2-4 points) and high probability groups (H2FPEF >5 points; HFA-PEFF >4 points). The primary outcome was a composite of all-cause mortality and hospitalizations due to HFpEF. Statistical significance was set at p<0.05.

**Results:**

A total of 103 patients were followed for an average of 888 days (±291). The mean age was 69 years (±8.3), and 61% were women. Twenty-seven patients (26.2%) experienced primary outcomes, totaling 32 events—11 deaths and 21 hospitalizations due to HFpEF. In the receiver operating characteristic (ROC) curve analysis, the H_2_FPEF score showed better predictive ability for the outcomes (area under the curve [AUC]: 0.637, 95% CI: 0.518–0.756, p=0.035) compared to the HFA-PEFF score (AUC: 0.572, 95% CI: 0.448–0.696, p=0.270). In the Kaplan-Meier analysis, high-probability classification by both scores was significantly associated with the occurrence of outcomes (log-rank p=0.034), compared to the intermediate score group or patients with differing classifications between the two scores.

**Conclusions:**

The H_2_FPEF score showed better performance than the HFA-PEFF score in predicting outcomes in patients with HFpEF. Findings from this contemporary study conducted in Brazil contribute to risk stratification in clinical practice.

## Introduction

Heart failure (HF) with preserved ejection fraction (HFpEF) is a common clinical syndrome associated with high rates of both cardiovascular and non-cardiovascular events. In Brazil, HF is the leading cause of hospital admissions and has a high readmission rate. Approximately half of the hospitalized patients have preserved ejection fraction.^1-7^

Diagnosing HFpEF remains a clinical challenge due to the high prevalence of comorbidities in this population and the nonspecific nature of its signs and symptoms, which may be triggered or worsened by those same comorbidities.^1,2^ In patients with signs and symptoms suggestive of chronic HF, along with risk factors and electrocardiographic changes, the diagnosis of HFpEF is based on a probabilistic approach that combines echocardiographic findings with the measurement of natriuretic peptides (e.g., N-terminal pro–B-type natriuretic peptide [NT-proBNP] or B-type natriuretic peptide [BNP]).

In about 30% to 35% of patients with HFpEF, exertional dyspnea is present even in the absence of clear signs of congestion at rest, either on physical examination or imaging.^3^ In such cases, diagnostic confirmation requires assessment of filling pressures during exercise, using stress echocardiography or cardiac catheterization.^3^ However, this approach is limited in clinical practice due to its complexity and high cost and is usually restricted to specialized centers.

Clinical scores such as H_2_FPEF and HFA-PEFF have been proposed as noninvasive tools to help identify patients with HFpEF.^3-9^ Based on probabilistic models that combine clinical and echocardiographic variables, such scores classify patients into low, intermediate, or high diagnostic probability, guiding the need for additional testing such as stress echocardiography or cardiac catheterization.

In addition to their diagnostic use, some studies have explored the application of these scores for prognostic assessment in patients with HFpEF.^5,6,9^ In the case of the H_2_FPEF score (an acronym for *Heavy, Hypertensive, Atrial Fibrillation, Pulmonary Hypertension, Elder, and Filling Pressures*), its individual components have each been associated with a higher risk of cardiovascular outcomes in various clinical studies. Similarly, the components of the HFA-PEFF score (an acronym for *Heart Failure Association Pre-test assessment, Echocardiography & natriuretic peptide, Functional testing, and Final etiology*) — such as indexed left ventricular mass, E/e’ ratio, indexed left atrial volume, pulmonary artery systolic pressure (PASP), and BNP—have also been linked to an increased risk of cardiovascular events.^6^

Recent studies involving patients diagnosed with HFpEF have shown that high scores on the H_2_FPEF (>5) and HFA-PEFF (>4) scales are associated with a higher risk of HF-related outcomes.9 However, there is a lack of contemporary prognostic studies conducted in Brazil in this population, as highlighted in a recent review.^9^

Therefore, this study aims to assess the prognostic value of current clinical scores in patients with HFpEF in a contemporary cohort in Brazil.

## Methods

### Study design and participants

This prospective cohort study was conducted at a tertiary university hospital with patients diagnosed with HFpEF. Between March 2019 and December 2021, participants were recruited in an outpatient setting, most of whom were already receiving cardiology follow-up at the institution. Patients included had been clinically stable for at least 1 month, were receiving optimized medical therapy according to current guidelines,^1^ and had clinically compensated comorbidities that did not contraindicate cardiopulmonary exercise testing (CPET).

### Inclusion criteria

HFpEF diagnostic criteria were assessed individually based on current literature recommendations.^1,2^ Patients were included if they met the following criteria: i) presence of HF symptoms and/or signs; ii) preserved left ventricular ejection fraction (LVEF) (>50%); iii) elevated natriuretic peptide levels (NT-proBNP >125 pg/mL and/or BNP >35 pg/mL) or structural abnormalities (increased indexed left ventricular mass or indexed left atrial volume) associated with diastolic dysfunction; and iv) absence of specific etiologies of HF with LVEF >50%, such as hypertrophic cardiomyopathy, restrictive cardiomyopathy, significant valvular disease, pericardial disease, and other specific causes of HFpEF.

To ensure a sample with a higher diagnostic probability of HFpEF, the H_2_FPEF score was calculated at the time of recruitment.4 Only patients who met the mandatory criteria for HFpEF and had either a high H2FPEF score (>5) or an intermediate score (2-5) with evidence of elevated filling pressures were included (Figure 1). The HFA-PEFF score was not used for screening as it was only published in October 2019.

**Figure 1:** – Flowchart of patient inclusion with HFpEF in the study.

Source: Prepared by the authors.

### Study protocol

Patients were invited to participate in the study through a phone call, during which they received a brief explanation of the study and were scheduled for a clinical interview with initial assessments. At that visit, detailed information about the study was provided, and written informed consent was obtained. Participants then underwent a brief clinical evaluation, including anthropometric measurements and bioimpedance analysis. Quality of life was assessed using the Minnesota Living with Heart Failure Questionnaire, in the Portuguese version translated and validated by Carvalho et al.^10^ Blood samples were then collected to measure NT-proBNP and/or BNP levels. Participants later returned to the clinic for echocardiography and CPET.

### Echocardiography

Images were acquired in 2-dimensional and M-mode, using color, continuous-wave, pulsed-wave, and tissue Doppler techniques. All examinations were performed using a Toshiba Aplio™ 300 system.

Quantitative measurements of the left ventricle were collected, including linear dimensions, left ventricular mass, and ejection fraction using the Simpson method. Left atrial volumes and diastolic function parameters were also assessed (E and A wave velocities, septal and lateral e’ velocities, E/e’ ratio, and estimated PASP). Right ventricular linear measurements and systolic function parameters were obtained as well, including tricuspid annular plane systolic excursion (TAPSE), S’ wave velocity, and fractional area change (FAC).

All assessments followed the recommendations of the *American Society of Echocardiography* guidelines for chamber quantification.^11^. In patients with atrial fibrillation (AF), mean values from 4-5 beats were used, selecting cycles within 20% of the average heart rate and with minimal variability in mitral inflow velocity, for the analysis of diastolic function and right ventricular systolic function.^12^

Images were recorded in DICOM format, exported to the electronic medical record, and later used for data extraction.

### Cardiopulmonary exercise testing

All tests were performed on a treadmill (GE T-2100, General Electric, USA) by the same investigators, using an individualized ramp protocol with progressively increasing workload, designed to be completed within 8 to 12 minutes. All participants underwent symptom-limited tests, following criteria for maximal effort.

Gas analysis was conducted using the Quark CPET system (COSMED, Rome, Italy), with real-time respiratory measurements, integrated with the OMNIA software (COSMED, Rome, Italy). Ventilatory thresholds (anaerobic threshold and respiratory compensation point) were determined using the ventilatory equivalents method, with an anaerobic threshold confirmed by the V-slope method.

The VE/VCO_2_ slope over the entire test and the *oxygen uptake efficiency slope* (OUES) were calculated. Predicted peak VO_2_ was estimated using the algorithm proposed by Wasserman and Hansen.

### H2FPEF and HFA-PEFF diagnostic scores

After completing all protocol assessments, the H_2_FPEF and HFA-PEFF scores were calculated for all patients, along with the diagnostic probability of HFpEF according to each scoring system.

The H2FPEF score combines clinical and echocardiographic variables, including obesity (*Heavy*), use of two or more antihypertensive medications (*Hypertensive*), Atrial Fibrillation, PASP >35 mmHg (*Pulmonary Hypertension*), age over 60 years (*Elder*), and E/e’ ratio >9 (*Filling Pressures*). Patients were classified according to the total score: >5 points (high probability), 2-5 points (intermediate probability), and 1 point (low probability) (Figure 2).

**Figure 2:** – H2FPEF score for HFpEF diagnosis and estimation of diagnostic probability.

Source: Adapted from Reddy et al.4

To calculate the HFA-PEFF score, echocardiographic variables from the morphological and functional domains were used, along with natriuretic peptide levels (NT-proBNP or BNP). These were stratified as major criteria (2 points) and minor criteria (1 point). Patients were classified according to the total score: >4 points (high probability), 2-4 points (intermediate probability), and 1 point (low probability) (Figure 3).

**Figure 3:** – HFA-PEFF score for HFpEF diagnosis and estimation of disease probability. Source: Adapted from Pieske B, et al. How to diagnose heart failure with preserved ejection fraction: the HFA-PEFF diagnostic algorithm: a consensus recommendation from the Heart Failure Association (HFA) of the European Society of Cardiology (ESC).7

### Primary outcome and follow-up

The primary outcome was defined as a composite of all-cause mortality and HF-related hospitalizations. Patients were prospectively followed for a minimum of 2 years and a maximum of 3 years through: i) active review of medical records, including outpatient visits and hospital admissions, and ii) periodic phone calls with participants to identify outcomes of interest. Individual outcome assessments were conducted every 6 months, and results were recorded in a standardized form and entered into REDCap software.^13^

### Statistical analysis

A sample size calculation was performed to detect a significant effect of peak oxygen consumption (peak VO2), categorized as above or below the median, using a Cox regression model. Equal-sized groups were considered, with the following estimates: a primary outcome rate of 8.83% in patients with VO2 >17.1 mL/kg/min and 31.17% in those with VO_2_ <17.1 mL/kg/min, with a hazard ratio (HR) of 3.53 over a 2-year follow-up.^14^ Calculation were performed using the “ssizeCT.default” function from the “powerSurvEpi” package in R software, version 3.5.0, assuming 80% statistical power and a 5% significance level. The estimated required sample size was 126 patients.

Statistical analyses were performed using SPSS software, version 29.0 (IBM SPSS Statistics, Chicago, IL, USA). The normality of variables was assessed using the Shapiro-Wilk test. Continuous variables with normal distribution were compared using the independent samples t-test, while non-normally distributed variables were analyzed using the Mann-Whitney test. Categorical variables were analyzed using the chi-square test.

Results were expressed as mean±standard deviation or median with interquartile ranges for continuous variables, and as absolute and relative frequencies for categorical variables. Cox proportional hazards regression was used to assess the independent and adjusted impact of variables on prognosis in a multivariable model. Receiver operating characteristic (ROC) curve analysis was used to compare the discriminatory ability of the scores in predicting outcomes. Survival analysis was performed using multivariable Cox regression and illustrated with Kaplan-Meier curves. Diagnostic agreement between the scores was assessed using the Kappa statistic. p-values <0.05 were considered statistically significant.

## Results

The final sample included 103 patients, with a mean age of 69.1 years (±8.3), and the majority were female (61.2%). Among the participants, 57 (55.3%) had a previous hospitalization for HF, and 82 (79.6%) were in *New York Heart Association* (NYHA) functional class I or II, while 21 (20.4%) were in class III. The mean follow-up time was 888 days (±291). The Central Illustration summarizes the main findings of the study.

After completing the protocol assessments, the H_2_FPEF and HFA-PEFF scores were calculated, and patients were classified into intermediate or high diagnostic probability groups for HFpEF. According to the H_2_FPEF score, 56 patients (54.3%) were classified as high probability and 47 (45.7%) as intermediate probability. According to the HFA-PEFF score, 61 patients (59.2%) had high probability, 41 (39.8%) intermediate probability, and 1 (1.0%) low probability. Considering the combination of both scores, 32 patients (31.1%) had high probability according to both, 53 (51.5%) had discordant results, and 18 (17.5%) were classified as intermediate probability by both scores. Agreement analysis between the scores showed a Kappa value of -0.036 (p=0.718).

The general characteristics of the sample, stratified according to H_2_FPEF and HFA-PEFF scores (intermediate or high probability), are presented in Tables 1 and 2. Values are expressed as means, medians, or relative frequencies, as appropriate.

**Table 1:** – Clinical and anthropometric characteristics according to H2FPEF and HFA-PEFF scores

Variable	H_2_FPEF intermediate (n=47)	H_2_FPEF high (n=56)	p-value	HFA-PEFF intermediate (n=41)	HFA-PEFF high (n=61)	p-value
**Age (years)**	68.6 (8.4)	69.4 (8.3)	0.632	69.0 (7.4)	69.4 (8.6)	0.804
**Female sex, n (%)**	33 (70%)	30 (54%)	0.106	27 (66%)	36 (59%)	0.537
**Prior HF hospitalizations, n (%)**	14 (30%)	43 (77%)	<0.001	26 (63%)	30 (49%)	0.223
**Comorbidities, n (%)**						
Obesity	36 (77%)	42 (75%)	1.000	33 (80%)	44 (72%)	0.360
Hypertension	47 (100%)	53 (95%)	0.248	39 (95%)	60 (98%)	0.563
Diabetes mellitus	30 (64%)	28 (50%)	0.170	23 (56%)	35 (57%)	1.000
Atrial fibrillation or flutter	3 (6%)	46 (82%)	<0.001	19 (46%)	29 (47%)	1.000
Coronary artery disease	19 (40%)	18 (32%)	0.415	12 (29%)	25 (41%)	0.295
Chronic kidney disease	20 (43%)	22 (39%)	0.841	18 (44%)	24 (39%)	0.685
**Lifestyle habits, n (%)**						
Physical activity	15 (32%)	12 (21%)	0.265	7 (17%)	19 (31%)	0.164
Smoking	26 (55%)	34 (61%)	0.689	23 (56%)	36 (59%)	0.839
**MLHFQ score**	33.2 (22.8)	33.4 (20.2)	0.969	35.9 (21.2)	31.5 (21.5)	0.325
**Medications in use, n (%)**						
Beta-blockers	43 (91%)	46 (82%)	0.249	35 (85%)	53 (87%)	1.000
ACE inibitors/ARB	36 (77%)	47 (84%)	0.454	33 (80%)	49 (80%)	1.000
Spironolactone	8 (17%)	8 (14%)	0.788	6 (15%)	9 (15%)	1.000
Loop diuretic	22 (47%)	39 (70%)	0.027	23 (56%)	37 (61%)	0.685
**Laboratory tests**						
Creatinine clearance (mL/min)	62.7 (19.4)	58.1 (17.8)	0.220	59.0 (20.9)	60.6 (16.9)	0.668
Hemoglobin (g/dL)	12.9 (1.4)	13.1 (1.6)	0.550	12.8 (1.5)	13.2 (1.5)	0.296
**Natriuretic peptides**						
NT-proBNP, median (pg/mL) (n=55)	225.7 (152.0-326.6)	695.1 (329.4-1.725.0)	<0.001	158.6 (121.2-308.3)	447.6 (246.4-1.318.2)	0.011
BNP, median (pg/mL) (n=28)	86.9 (31.5-123.9)	147.4 (118.1-273.2)	0.002	70.2 (23.5-144.0)	126.8 (89.2-190.4)	0.035
**Anthropometric measurements**						
BMI (kg/m2)	34.0 (5.7)	33.3 (5.3)	0.529	34.4 (6.0)	33.1 (5.1)	0.249
Lean mass (%)	58.2 (8.5)	62.2 (12.1)	0.054	58.9 (10.6)	61.3 (10.9)	0.277

For continuous variables, Student’s t-test or the Mann-Whitney test was used, depending on data distribution. For categorical variables, the chi-square test was applied. Dispersion measures are shown in parentheses as SD or IQR, as appropriate. ARB: angiotensin receptor blocker; SD: standard deviation; AF: atrial fibrillation; ACE: angiotensin-converting enzyme; IQR: interquartile range; BMI: body mass index; MLHFQ: Minnesota Living with Heart Failure Questionnaire.

Source: Prepared by the authors.

**Table 2:** – Echocardiographic and cardiopulmonary exercise testing results according to H2FPEF and HFA-PEFF scores

Variable	H_2_FPEF intermediate (n=47)	H_2_FPEF high (n=56)	p-value	HFA-PEFF intermediate (n=41)	HFA-PEFF high (n=61)	p-value
**Echocardiography**						
LV mass (g/m^2^)	92.4 (24.0)	98.2 (22.1)	0.207	90.8 (21.6)	98.9 (23.7)	0.082
Septal wall thickness (mm)	10.5 (1.5)	10.9 (1.5)	0.174	10.7 (1.5)	10.7 (1.6)	0.804
Posterior wall thickness (mm)	10.0 (1.4)	10.3 (1.6)	0.311	10.0 (1.5)	10.3 (1.5)	0.365
RWT	0.43 (0.07)	0.43 (0.07)	0.761	0.44 (0.08)	0.43 (0.06)	0.624
Ejection fraction (%)	61.5 (4.4)	58.4 (4.3)	0.001	58.9 (5.2)	60.5 (4.0)	0.090
Indexed LA volume (mL/m^2^)	41.8 (9.8)	53.8 (16.0)	<0,001	45.3 (12.8)	50.8 (15.4)	0.061
Indexed RA volume (mL/m^2^)	31.1 (15.2)	44.4 (20.9)	<0,001	34.5 (14.7)	41.5 (22.4)	0.082
RV basal diameter (mm)	36.5 (3.8)	39.4 (5.3)	0.002	38.8 (4.6)	37.4 (5.0)	0.157
TAPSE (mm)	20.2 (3.9)	17.3 (4.0)	<0,001	17.9 (4.2)	19.1 (4.1)	0.147
FAC (%)	44.4 (5.6)	42.2 (5.8)	0.069	43.1 (6.6)	43.3 (5.2)	0.879
PASP (mmHg)	28.6 (5.1)	37.3 (10.7)	<0,001	32.9 (8.6)	35.2 (10.8)	0.310
Central venous pressure (mmHg)	4.5 (2.3)	6.9 (4.3)	<0,001	5.8 (3.4)	5.8 (4.0)	0.974
Septal e’ wave (cm/s)	5.7 (1.4)	6.9 (2.0)	0.001	6.6 (2.3)	6.2 (1.5)	0.310
Lateral e’ wave (cm/s)	7.1 (1.8)	9.3 (2.9)	<0.001	9.0 (2.7)	7.8 (2.5)	0.020
E/e’ ratio	13.1 (4.7)	13.2 (6.2)	0.926	12.5 (5.6)	13.6 (5.4)	0.323
**Cardiopulmonary exercise testing**						
Peak HR (% of predicted)	81.5 (12.3)	85.7 (17.1)	0.159	87.0 (13.6)	81.3 (15.7)	0.071
Peak VO_2_ (mL/kg/min)	15.1 (2.5)	14.7 (3.2)	0.465	14.4 (2.9)	15.1 (2.9)	0.245
Peak VO_2_ (% of predicted)	80.5 (14.8)	74.0 (15.2)	0.033	75.4 (12.9)	78.2 (16.8)	0.380
VE/VCO_2_ slope	33.2 (7.7)	38.5 (8.8)	0.002	35.7 (9.0)	36.3 (8.6)	0.723
Peak VE/VCO_2_	32.5 (6.5)	36.9 (5.5)	0.001	34.9 (7.0)	34.6 (6.0)	0.827
PetCO_2_ (mmHg)	32.4 (4.4)	30.8 (4.2)	0.085	32.5 (4.2)	30.9 (4.4)	0.088

Student’s t-test was used for continuous variables. Values are presented as mean (SD). RA: right atrium; LA: left atrium; SD: standard deviation; RWT: relative wall thickness; FAC: fractional area change; HR: heart rate; LV: left ventricular; PASP: pulmonary artery systolic pressure; TAPSE: tricuspid annular plane systolic excursion; RV: right ventricle; VO2: oxygen consumption.

Source: Prepared by the authors.

### H2FPEF and HFA-PEFF diagnostic scores

The results are presented in Table 3. In 20 patients (19.4%), PASP could not be technically estimated. In another 20 patients (19.4%), natriuretic peptide levels were not collected due to changes in the institutional protocol, which were only resolved shortly before the start of the pandemic, making timely sample collection unfeasible.

**Table 3:** – Clinical and echocardiographic variables included in the H2FPEF and HFA-PEFF scores

Variables	Intermediate score	High score	p-value
**H_2_FPEF score (n=103)**	47 (46%)	56 (54%)	
Total score	4.3 (0.9)	7.3 (1.1)	<0.001
Obesity	35 (74%)	42 (75%)	1
Use of ≥2 antihypertensives	44 (94%)	49 (87%)	0.339
AF	3 (6%)	46 (82%)	<0.001
Age >60 years	38 (81%)	49 (87%)	0.419
E/e’ ratio >9	40 (85%)	40 (71%)	0.153
PSAP >35 mmHg	3 (6%)	32 (57%)	<0.001
PASP unavailable	16 (34%)	4 (7%)	0.001
**HFA-PEFF score (n=102)**	41 (40%)	61 (59%)	
Total score	3.6 (0.7)	5.7 (0.5)	<0.001
Functional domain*			
Septal e’ <7 cm/s	27 (66%)	44 (72%)	0.518
Lateral e’ <10 cm/s	29 (71%)	49 (80%)	0.342
Average E/e’ >15	9 (22%)	19 (31%)	0.369
Average E/e’ 9-14	20 (49%)	32 (52%)	0.840
TR velocity >2.8 m/s	13 (32%)	23 (38%)	0.673
TR velocity unavailable	5 (12%)	15 (25%)	0.137
**Morphological domain**			
LA volume >34 mL/m2	34 (83%)	58 (95%)	0.085
LA volume 29-34 mL/m2	3 (7%)	3 (5%)	0.682
LV mass >149/122 g/m2 (M/F) + RWT >0.42	1 (2%)	5 (8%)	0.397
LV mass >115/95 g/m2 (M/F)	10 (24%)	22 (36%)	0.278
RWT >0.42	19 (46%)	34 (56%)	0.420
Wall thickness >12 mm	8 (19%)	17 (28%)	0.360
**Natriuretic peptides**			
NT-proBNP >220 / BNP >80 (without AF)	0 (0%)	33 (54%)	<0.001
NT-proBNP 125–220 / BNP 35–80 (without AF)	2 (5.0%)	9 (15%)	0.192
NT-proBNP >660 / BNP >240 (with AF)	2 (5.0%)	13 (21%)	0.024
NT-proBNP 365–660 / BNP 105–240 (with AF)	1 (2%)	6 (10%)	0.237
Did not meet criteria	16 (39%)	0 (0%)	<0.001
Peptide not collected	20 (49%)	0 (0%)	<0.001

*The global longitudinal strain (GLS) variable was not included in this study. The chi-square test was used for categorical variables. LA: left atrium; BNP: B-type natriuretic peptide; RWT: relative wall thickness; AF: atrial fibrillation; M/F: male/female; LV: left ventricular mass; PASP: pulmonary artery systolic pressure; TR: tricuspid regurgitation.

Source: Prepared by the authors.

### Primary outcomes

Of the 103 patients, 27 (26.2%) experienced primary outcomes, totaling 32 events — 11 deaths and 21 HF-related hospitalizations. Among the deaths, four were due to cancer-related complications, three to respiratory sepsis, three to cardiovascular causes, and one to COVID-19. The mean time to the first event was 563 days (±356). The results are presented in Table 4.

**Table 4:** – Primary outcomes and prognostic scores according to H2FPEF and HFA-PEFF scores

Variable	Total HFpEF (n=103)	H2FPEF intermediate (n=47)	H2FPEF high (n=56)	p-value	HFA-PEFF intermediate (n=41)	HFA-PEFF high (n=61)	p-value
**Patients with primary outcomes, n (%)**	27 (26%)	7 (15%)	20 (36%)	0.024	8 (19%)	19 (31%)	0.254
H_2_FPEF score	6.5 (1.6)	4.4 (0.8)	7.3 (1.1)		6.6 (1.7)	6.5 (1.6)	
HFA-PEFF score	5.2 (1.0)	5.4 (1.0)	5.1 (1.1)		3.8 (0.5)	5.8 (0.4)	
**Primary outcome events, n**	32	10	22		9	23	
Death, n (%)	11 (11%)	5 (11%)	6 (11%)	0.990	3 (7.3%)	8 (13%)	0.518
HF hospitalizations, n (%)	21 (20%)	5 (11%)	16 (29%)	0.029	6 (15%)	15 (25%)	0.318
MAGGIC score (mean±SD)	17.3 (4.7)	16.9 (4.5)	17.7 (5.0)	0.420	17.3 (4.6)	17.5 (4.7)	0.800

Percentages in parentheses refer to the total number of patients (n) in each column. SD: standard deviation; HF: heart failure; HFpEF: heart failure with preserved ejection fraction; MAGGIC: Meta-Analysis Global Group in Chronic Heart Failure.

Source: Prepared by the authors.

Patients with a high H_2_FPEF score had a higher frequency of primary outcomes compared to those with an intermediate score (35.7% vs 14.9%; p<0.024) as well as a greater occurrence of HF-related hospitalizations (29.0% vs 11.0%; p<0.029). In contrast, for the HFA-PEFF score, there was no statistically significant difference between the intermediate and high probability groups.

When both criteria were combined, patients with high scores on both the H_2_FPEF and HFA-PEFF scales had a higher frequency of outcomes compared to those with discordant scores or intermediate scores on both (43.8% vs 20.7% vs 11.1%; p<0.007). The results are presented in Table 5.

**Table 5:** – Primary outcomes according to combined H2FPEF and HFA-PEFF scores

Variables	Total HFpEF (n=103)	Both intermediate (n=18)	Discordant scores (n=53)	Both high (n=32)	p-value
**Patients with primary outcome, n (%)**	27 (26%)	2 (11%)	11 (20,7%)	14 (44%)	<0.007
H_2_FPEF score	6,5 (1,6)	4,5 (0,7)	6,0 (1,84)	7.3 (1,1)	
HFA-PEFF score	5,2 (1,0)	4,0 (0,0)	4,7 (1,3)	5.7 (0,4)	
**Total primary outcome events, n**	32	3	13	16	
Death, n (%)	11 (11%)	2 (11%)	4 (7,5%)	5 (16%)	0.491
HF hospitalizations, n (%)	21 (20%)	1 (6%)	9 (17,0%)	11 (34%)	0.012

Percentages in parentheses refer to the total number of patients (n) in each column. HF: heart failure; HFpEF: heart failure with preserved ejection fraction.

Source: Prepared by the authors.

### Outcome predictors

In the Cox regression analysis, the univariate model showed that a high H_2_FPEF score was not a statistically significant predictor of events (HR: 2.316; 95% CI: 0.973-5.513; p=0.058), nor was a high HFA-PEFF score (HR: 1.570; 95% CI: 0.683-3.611; p=0.288). The simultaneous presence of high scores on both the H2FPEF and HFA-PEFF scales was also not significantly associated with outcome prediction (HR: 3.850; 95% CI: 0.868-17.071; p=0.076).

In the ROC curve analysis, the H2FPEF score showed better predictive ability for outcomes (AUC: 0.637; 95% CI: 0.518-0.756; p=0.035) compared to the HFA-PEFF score (AUC: 0.572; 95% CI: 0.448-0.696; p=0.270). When both scores were combined, there was an improvement in discriminatory power for predicting outcomes (AUC: 0.662; 95% CI: 0.543-0.782; p=0.013). Results are shown in Figure 4.

**Figure 4:** – ROC curves of the H2FPEF, HFA-PEFF, and for outcome prediction.

Source: Prepared by the authors.

In the survival analysis using the Kaplan-Meier method, a high H_2_FPEF score showed a trend toward statistical significance in predicting outcomes (log-rank p=0.05), while the HFA-PEFF score was not significant (log-rank p=0.284). The simultaneous presence of high scores on both criteria was significantly associated with the occurrence of outcomes (log-rank p=0.034), compared to the combined group of patients with discordant scores or intermediate scores on both scales. Figure 5 shows the Kaplan-Meier survival curves according to each score evaluated.

**Figure 5:** – Kaplan-Meier survival curves according to the HFA-PEFF score (A), H2FPEF score (B), and combined scores (C).

Source: Prepared by the authors.

## Discussion

This study aimed to evaluate the prognostic impact of the H2FPEF and HFA-PEFF clinical scores in a well-characterized sample of patients with HFpEF in Brazil. Both scores were developed to support the noninvasive diagnosis of HFpEF by stratifying patients into low, intermediate, and high diagnostic probability groups (Figure 6). The results showed that the H2FPEF score had better performance than the HFA-PEFF score in predicting cardiovascular outcomes in a prospective cohort of patients with HFpEF. Moreover, the combination of both scores was associated with an increased risk of cardiovascular events. Given the high prevalence of HFpEF in Brazil, these findings have clinical relevance as they allow for the noninvasive identification of patients at higher risk of serious cardiovascular events.

**Figure 6:** - Current probabilistic diagnostic model for HFpEF.

Source: Marcondes-Braga FG et al. Emerging Topics Update of the Brazilian Heart Failure Guideline – 2021.^8^

The H_2_FPEF score was developed based on a cohort of patients with an invasive diagnosis of HFpEF confirmed by exercise right heart catheterization and later validated in a second cohort that included both patients with and without HFpEF.4 The clinical variables included in the score—obesity, atrial fibrillation, use of two or more antihypertensive medications, age over 60 years, E/e’ ratio >9, and PASP >35 mmHg — were shown to be predictors of cardiovascular outcomes in a multivariable model. High H_2_FPEF scores (>5) were associated with a diagnostic probability of HFpEF greater than 90%. The HFA-PEFF score, in turn, was developed through a consensus by the European Society of Cardiology and is based on a four-step sequential diagnostic approach: i) initial pre-test assessment; ii) probabilistic evaluation based on echocardiographic findings and natriuretic peptide levels; iii) specialized confirmatory testing; and iv) etiological assessment.7 In the second step of the algorithm, the cutoff values for echocardiographic variables (morphological and functional domains) and NT-proBNP/BNP levels were defined based on their sensitivity and specificity for diagnosing HFpEF, as supported by clinical evidence. These variables were classified as major (2 points) or minor (1 point) criteria. HFA-PEFF scores greater than 4 were defined by the consensus as indicative of high diagnostic probability of HFpEF. Unlike the H_2_FPEF score, the HFA-PEFF criteria were not initially validated in a cohort of patients with and without HFpEF.7,15,16

Reflecting the clinical profile commonly seen in patients with HFpEF, our sample was composed of older individuals, mostly women, with obesity, hypertension, diabetes, chronic kidney disease, and coronary artery disease. The prevalence of atrial fibrillation and other cardiac abnormalities—such as left ventricular hypertrophy, diastolic dysfunction, left atrial enlargement, pulmonary hypertension, and right ventricular dysfunction — was consistent with findings from a recent clinical review on patients with HFpEF.^3^

In the present study, at the beginning of the enrollment phase (March 2019), the H_2_FPEF score was used to noninvasively estimate the diagnostic probability of HFpEF. Only patients with a high score or an intermediate score combined with evidence of elevated filling pressures were included. The sample consisted of a substantial proportion of patients with a high diagnostic probability: 54% based on the H_2_FPEF score and 59% based on the HFA-PEFF score. These rates are higher than those reported in recent prognostic studies that also evaluated the application of these scores. In the study by Przewlocka-Kosmala et al.,^5^ 30% of patients had an H_2_FPEF score >5 and 41% had an HFA-PEFF score >4. In the study by Egashira et al.,6 38% of patients had an HFA-PEFF score >4.

Among the intermediate and high score groups based on the HFA-PEFF criteria, the only statistically significant difference was observed in natriuretic peptide levels. The same pattern was seen in the stratification using the H_2_FPEF score, even though this parameter is not included in its calculation. According to a recent study by Reddy et al.,^17^ the differences in diagnostic accuracy between the H_2_FPEF and HFA-PEFF scores may be related to the primary components of each model. The H_2_FPEF score includes clinical variables such as obesity, hypertension, and AF, which increase the pre-test probability of HFpEF. In contrast, the HFA-PEFF score relies on echocardiographic findings and natriuretic peptide levels — parameters that are less sensitive for diagnosis, although still informative regarding disease presence. In patients with atrial fibrillation, the HFA-PEFF score applies higher cutoff values for natriuretic peptides to confirm the diagnosis of HFpEF.^17^

In the present study, a higher rate of primary outcomes was observed among patients with a high diagnostic probability compared to those with intermediate probability, with statistical significance reached only for the H_2_FPEF score and for the combined scores. Patient stratification into intermediate and high probability groups using the HFA-PEFF score was limited to step 2 of the algorithm proposed by the consensus, as no echocardiographic assessments during exercise (step 3) were performed. As demonstrated by Przewlocka-Kosmala et al.,^5^ incorporating stress echocardiography may enhance the predictive value of the HFA-PEFF score.

In the present sample, combining high-probability scores improved the identification of cardiovascular outcomes. However, as shown in other studies,^5-7,18-20^ there was significant disagreement in diagnostic probability estimates when both scores were used together. Divergent classifications were observed in 51% of patients, with agreement found in only 31% of those with high scores and 17% of those with intermediate scores. This variability limits the clinical applicability of the combined approach in routine care.

Our study has some limitations. The most relevant was the onset of the COVID-19 pandemic during the enrollment period, which had to be interrupted in March 2020 and was resumed only in April 2021 under more restrictive conditions due to isolation and prevention measures. This interruption made it impossible to reach the planned sample size (n=126), even with an 18-month extension of the inclusion period. Nevertheless, all enrolled patients (n=103) completed the minimum 2-year and maximum 3-year follow-up as established in the protocol. Only one death was related to COVID-19, so the generalizability of the results was not compromised. It is possible that some additional outcomes were not recorded due to incomplete follow-up in a small portion of the sample (n=5). Second, during the study, there was an institutional change in the type of natriuretic peptide used (from NT-proBNP to BNP). Until sample collection was standardized—shortly before the pandemic began—this change resulted in missing data for 20 patients. Since BNP/NT-proBNP levels are included in the calculation of the HFA-PEFF score, this limitation may have affected the accuracy of risk stratification between the intermediate and high probability groups. Third, in 20 patients (19.4%), PASP could not be estimated by echocardiography — a variable used in the H2FPEF score. Although this percentage is lower than those reported in other studies (30-40%),^18,21,22^ the unavailability of such data may have introduced some inaccuracy in the classification between intermediate and high risk in this score. Even so, our sample showed similar proportions of patients with high diagnostic probability according to both scores (54% for H_2_FPEF and 59% for HFA-PEFF), which allowed for consistent group comparisons. Fourth, the absence of stress echocardiography may have limited the predictive ability of the HFA-PEFF score, as no significant differences were observed between the intermediate and high-risk groups using step 2 of the diagnostic algorithm. However, this modality was not available at our hospital, is costly, and is limited to a few specialized centers, which also restricts its use in clinical practice. Finally, this was a single-center study, which may limit the generalizability of the findings to other populations. Nevertheless, the cohort analyzed is representative of patients with HFpEF, as described in a recent clinical review.^3^

Among the main strengths of the present study, the first is its prospective cohort design, with the inclusion of patients based on specific and updated clinical criteria for the diagnosis of HFpEF, accurately reflecting the population affected by the disease. Second, the study applied a noninvasive clinical protocol focused on HFpEF, which included a brief clinical evaluation, anthropometric and bioimpedance measurements, natriuretic peptide testing, echocardiography, and exercise testing. This protocol supports comparisons with other populations and enhances the generalizability of the findings. Third, the results obtained hold clinical relevance for national health care practice because of the scarcity of contemporary prognostic studies involving patients with HFpEF in Brazil.

## Conclusions

In a prospective cohort of patients with HFpEF in Brazil, the H_2_FPEF score showed superior performance compared to the HFA-PEFF score in predicting cardiovascular outcomes. The combination of both scores, when indicating high probability, provided greater prognostic value for cardiovascular events. Given the high prevalence of HFpEF in the country, these findings are clinically relevant as they enable the noninvasive identification of patients at higher risk of serious cardiovascular outcomes.
